# Antibacterial Evaluation and Virtual Screening of New Thiazolyl-Triazole Schiff Bases as Potential DNA-Gyrase Inhibitors

**DOI:** 10.3390/ijms19010222

**Published:** 2018-01-11

**Authors:** Cristina Nastasă, Dan C. Vodnar, Ioana Ionuţ, Anca Stana, Daniela Benedec, Radu Tamaian, Ovidiu Oniga, Brînduşa Tiperciuc

**Affiliations:** 1 Department of Pharmaceutical Chemistry, “Iuliu Haţieganu” University of Medicine and Pharmacy, 41 Victor Babeş Street, RO-400012 Cluj-Napoca, Romania; ionut.ioana@umfcluj.ro (I.I.); teodora_anca@yahoo.com (A.S.); onigao65@yahoo.com (O.O.); brandu32@yahoo.com (B.T.); 2 Department of Food Science and Technology, University of Agricultural Sciences and Veterinary Medicine, 3-5 Mănăştur Street, RO-400372 Cluj-Napoca, Romania; 3 Department of Pharmacognosy, “Iuliu Haţieganu” University of Medicine and Pharmacy, 12 Ion Creangă Street, RO-400010 Cluj-Napoca, Romania; dani_67ro@yahoo.com; 4 National Institute for Research and Development for Cryogenic and Isotopic Technologies, 4th Uzinei Street, RO-240050 Râmnicu Vâlcea, Romania; 5 SC Biotech Corp SRL, 4th Uzinei Street, RO-240050 Râmnicu Vâlcea, Romania

**Keywords:** Schiff base, thiazole, triazole, antibacterial activity, ADMET, molecular docking, DNA-gyrase

## Abstract

The global spread of bacterial resistance to drugs used in therapy requires new potent and safe antimicrobial agents. DNA gyrases represent important targets in drug discovery. Schiff bases, thiazole, and triazole derivatives are considered key scaffolds in medicinal chemistry. Fifteen thiazolyl-triazole Schiff bases were evaluated for their antibacterial activity, measuring the growth inhibition zone diameter, the minimum inhibitory concentration (MIC), and the minimum bactericidal concentration (MBC), against Gram-positive (*Staphylococcus aureus*, *Listeria monocytogenes*) and Gram-negative (*Escherichia coli*, *Salmonella typhimurium*, *Pseudomonas aeruginosa*) bacteria. The inhibition of *S. aureus* and *S. typhimurium* was modest. Compounds **B1**, **B2**, and **B9** showed a similar effect as ciprofloxacin, the antimicrobial reference, against *L. monocytogenes*. **B10** displayed a better effect. Derivatives **B1**, **B5**–**7**, **B9**, and **B11**–**15** expressed MIC values lower than the reference, against *L. monocytogenes*. **B5**, **B6**, and **B11**–**15** strongly inhibited the growth of *P. aeruginosa*. All compounds were subjected to an in silico screening of the ADMET (absorption, distribution, metabolism, elimination, toxicity) properties. Molecular docking was performed on the gyrA and gyrB from *L. monocytogenes*. The virtual screening concluded that thiazolyl-triazole Schiff base **B8** is the best *drug-like* candidate, satisfying requirements for both safety and efficacy, being more potent against the bacterial gyrA than ciprofloxacin.

## 1. Introduction

The alarming worldwide spread of the bacterial resistance, to most of the drugs available nowadays in therapy [1,2,3], urgently requires the development of new effective antibacterial agents.

The DNA topoisomerases manage the topological state of the DNA in the cell, being involved in replication, transcription, recombination, and chromatin remodeling. In the bacterial proteome, there are various types of topoisomerases, the most common being type I topoisomerases, type II topoisomerases (DNA gyrases), and type IV topoisomerases [4].

One of the major targets of antibacterial compounds is represented by DNA gyrase (Type II topoisomerase), an enzyme playing an essential role in bacterial replication [5]. Structurally, the DNA gyrase is built of two A subunits (gyrA) and two B subunits (gyrB) that form an A2B2 heterotetramer. Topoisomerase IV consists of two constitutive subunits: parE (homologous to DNA gyrase subunit B–gyrB) and parC (homologous to DNA gyrase subunit A–gyrA) [6]. DNA gyrase has four functional domains (GO (Gene Ontology) terms) [7,8]. Domain 1, the N-terminal of domain gyrB, harbors the ATPase activity. The type II topoisomerases use the hydrolysis of ATP, in the presence of Mg^2+^, to simultaneously cut both strands of the double-helix, in order to manage DNA tangles and supercoils. Domain 2, the C-terminal domain of gyrB, which consists of a Toprim structural motif and a tail region, contributes to the binding of DNA, via the interaction with the gyrA subunit [9,10]. Domain 3, the N-terminal domain of gyrA, is responsible for the breaking-rejoining function, through its capacity to form protein-DNA bridges; meanwhile, Domain 4, the C-terminal domain of gyrA (TOP4c), is able to non-specifically bind DNA [11]. The Toprim domain (also known as the Rao-Rossmann fold) is a structural motif found in DNA primases, topoisomerases, and some enzymes involved in phosphotransfers or able to hydrolyze phosphodiester bonds [10]. The central DNA-binding core of gyrA contains the active site tyrosine residues located in the catabolite-activator-protein-like (CAP-like) domain which includes the DNA binding helix-turn-helix (HTH) motif. The CAP-like tyrosine residues are crucial for the breakage and religation of the DNA, by forming an ester with the 5′phosphate of the DNA [12]. The Toprim domain of subunit B is adjacent to the catalytic tyrosine in the CAP domain of subunit A and both form an active site, essential for the DNA-cleavage [10].

Quinolones are the only class of DNA gyrase inhibitors that are clinically used. Their effect is based on the inhibition of the gyrA subunit, therefore perturbing the DNA cleavage and the introduction of negative supercoils into the bacterial DNA [13]. Cyclothialidines [14] and aminocoumarins [15] are studied for their inhibition of the ATP-binding site of the gyrB subunit. Because of the bacterial resistance to fluoroquinolones used in therapy and also of their side effects and limitations, there are gyrase inhibitors searched, from different chemical classes: benzimidazoles, benzoxazole, benzothiazole, oxazolopyridines [16], aminopyrazinamides [17], thiazole derivatives [18,19,20], and triazole derivatives [21,22], which differently bind the biological target.

Schiff bases are a group of compounds which have gained popularity as biologically active scaffolds due to their ease of synthesis, their versatility, and their large spectra of activities, such as their antimicrobial [23,24], anti-inflammatory, anticonvulsant, and antioxidant [25] properties. Schiff bases have sufficient water solubility and are stable in vitro and in vivo [26]. The imine bond in Schiff bases provides binding opportunities with different nucleophiles and electrophiles, inhibiting enzymes or DNA replication. The isosteric replacement of the quinolones’ 3-carboxyl group, essential for gyrase binding, with an amino-thiazolic fragment [27] or other azoles (thiazoles, triazoles), led to molecules with improved antimicrobial effects and a wider spectrum of activity. These compounds express fewer side effects and have a better capacity in overcoming bacterial resistance [28,29].

Even if research efforts are continuously made, there still remains the challenge to discover new drugs of a high potential, large spectrum of activity, and with a good safety profile. As a continuation of our efforts in discovering new heterocyclic Schiff bases with potent antimicrobial properties [30], we synthesized a series of new Schiff bases of thiazolyl-triazole [31]. Prompted by the aspects described above, the work presented here focuses on the investigation of the antibacterial potential of the new azolyl-Schiff bases against Gram-positive and Gram-negative bacteria. A molecular docking study was realized on DNA gyrases of *Listeria monocytogenes*. ADMET profiling for risks and safety risks was also conducted for the new series of compounds.

## 2. Result and Discussion

### 2.1. Antibacterial Activity

#### 2.1.1. Determination of the Inhibition Zone Diameters

**Figure 1:**
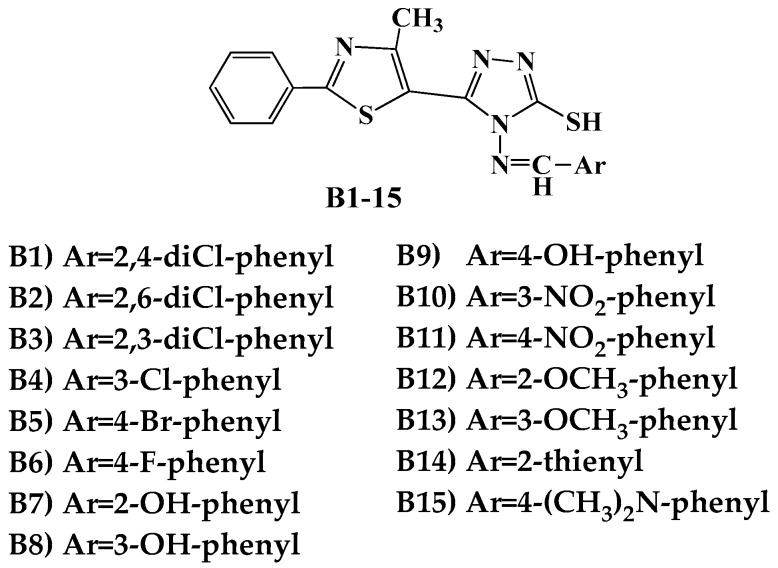
Structures of the Schiff bases **B1**–**15**.

The antimicrobial activity was tested in vitro using the disk diffusion method, by measuring the diameters of the inhibition zones. The synthesized compounds **B1**–**15** (Figure 1) [31] were screened against two Gram-positive (*Listeria monocytogenes* ATCC 35152, *Staphylococcus aureus* ATCC 25923) and three Gram-negative (*Escherichia coli* ATCC 25922, *Salmonella typhimurium* ATCC 13311, *P. aeruginosa* ATCC 27853) bacterial strains (Table 1).

For evaluating the antimicrobial activity, 100 µg/disk of the synthesized compounds and also of the reference substance, ciprofloxacin, were used. The solvent for the preparation of the solutions, dimethylsulfoxide (DMSO), exhibited no inhibitory activity on the bacterial strains considered for this study.

Regarding the activity against the Gram-positive bacteria, the strain of *Listeria monocytogenes* was more sensible to the tested compounds, three of them (**B1**, **B2**, and **B9**) showing a similar effect to ciprofloxacin, with an 18 mm inhibition zone and an AI value of 100%. **B10** (3-nitro-phenyl) proved to be more active than the antibacterial reference, displaying a 20 mm diameter and an AI of 111.1%. The second Gram-positive bacterium, *Staphylococcus aureus*, was moderately inhibited by the new molecules, with inhibition zones ranging from 12 to 18 mm, respectively, and AI between 42.8 and 64.2%. In the case of both strains, compound **B10** exhibited the most pronounced effect.

The inhibitory activity against *Escherichia coli*, *Salmonella typhimurium*, and *Pseudomonas aeruginosa* was modestly related to ciprofloxacin. The compounds determined zones of 14–16 mm (AI 51.8–59.2%) against *Escherichia coli*. The values registered on *Salmonella typhimurium* ranged between 16–18 mm (AI 72.7–81.8%). The percentage activity index against *Pseudomonas aeruginosa* was between 61.5%, corresponding to a 16 mm inhibitory zone diameter, and 80.7%, corresponding to a 21 mm zone diameter. The most potent derivatives were **B5**, **B6**, and **B11**–**15**.

From the results obtained, it can be observed that Schiff base **B10** expressed the most pronounced antibacterial effect.

#### 2.1.2. Determination of MIC and MBC Values

The broth microdilution method was employed for the minimum inhibitory concentration test. All synthesized compounds were tested against two Gram-positive bacterial strains (*Staphylococcus aureus* ATCC 49444, *Listeria monocytogenes* ATCC 19115) and two Gram-negative bacterial strains (*Pseudomonas aeruginosa* ATCC 27853, *Salmonella typhimurium* ATCC 14028). Stock solutions (1 mg/mL) were prepared by dissolving the test compounds and the reference antimicrobial, ciprofloxacin, in sterile DMSO. The results are presented in Table 2 (MIC and MBC).

Analyzing the results obtained, it can be seen that the growth inhibitory activity against the Gram-positive bacteria was more pronounced against the strain of *Listeria monocytogenes*, where 10 of the compounds (**B1**, **B5**–**7**, **B9**, **B11**–**15**) expressed MIC values lower than that of ciprofloxacin. The others showed the same effect as the reference. The strain of *Staphylococcus aureus* was less sensitive to the activity of the new molecules.

Growth of *Pseudomonas aeruginosa* was strongly inhibited by most of the compounds. It can be observed that **B5**, **B6**, and **B11**–**15** had MICs lower than the antibacterial used as the reference, while **B9** had the same active concentration, in agreement with the inhibitory zone diameters. The inhibition of *Salmonella typhimurium* was modest for all the tested derivatives.

Determination of MBC confirmed the results previously obtained, when MIC was investigated. The MBC value of Schiff base **B8** (meta-hydroxy) against *L. monocytogenes* ATCC 19115 was inferior to that of ciprofloxacin. For this compound, MIC was equal to the antibacterial reference. Also, the MBC of 1.95 µg/mL should be noted, registered for **B12** (ortho-methoxy-phenyl), against the strain of *P. aeruginosa*, which is much smaller than that for ciprofloxacin. The MBC/MIC ratio (Table S1) is one or two for all the tested compounds, suggesting that they may exert bactericidal activity [32,33].

### 2.2. Virtual Screening

Virtual screening (VS) emerged as an adaptive response of cheminformatics to organic chemistry requirements, in order to prioritize the synthesis of the most promising drug candidates [34,35,36,37]. In this respect, various cheminformatics tools can be used to filter the candidate compounds, based on their absorption, distribution, metabolism, excretion, toxicity (ADMET) [38,39,40], and their spatial interaction with the targets and their binding affinity (BA) via molecular docking [40,41]. Development of novel antimicrobials as DNA gyrase inhibitors (validated drugs targets) has been exploited in both computational and wet-lab strategies [5,20,42].

In our VS setup, we assessed the activity and potency of the newly synthesized Schiff bases, via molecular docking, on the two subunits of topoisomerases II (gyrA and gyrB) from *Listeria monocytogenes*, comparing the results with ciprofloxacin, chosen as the reference drug. We also evaluated the safety-related concerns by the means of ADMET profiling. An academic license of MarvinSketch was used for the drawing and displaying of 2D structures, 3D optimization of all ligands, and also for generating the input SDF files for ADMET profiling and Tripos MOL2 files for docking, MarvinSketch 17.6.0, 2017, ChemAxon, Budapest, Hungary [43].

#### 2.2.1. ADMET Profiling

ADMET profiling provides helpful guidance on the absorption, plasma clearance, tissue distribution, metabolic effects, and both acute and later toxicity. The results of the ADME study previously conducted and presented [31] showed that the thiazolyl-triazole Schiff bases **B1**–**15** displayed good pharmakocinetic properties and that all new molecules passed the drug-likeness criteria.

Toxicity studies are mandatory for a new product, due to the fact that a drug has to not only manifest its efficacy, but also have good tolerability and a low toxicity rate. Considering the above aspects as a good starting point, we continued the analysis of the newly synthesized compounds (Table 3).

The risks and safety profiling of the investigated compounds (Table 3) indicates that all compounds are non-inducers of phospholipidosis. On the other hand, ciprofloxacin (**CIP**) was classified as not PPI friendly, despite its approved drug status; meanwhile, all the Schiff bases successfully complied with this safety criterion. Referring to the problematic moieties, **CIP** is free of covalent inhibitors; meanwhile, all Schiff bases have in their framework structure the thiol (–SH) group, which is considered responsible for covalent binding [44,45]. Moreover, all compounds contain at least one low risk UMSs substructure: a halogenure in the case of **CIP**, respectively the aforementioned –SH group in the case of Schiff bases. Compounds **B1**–**6** also have a secondary low risk halogenure. Meanwhile, other low risk groups are present as follows: phenol (**B7**–**9**), nitro (**B10**–**11**), and thiophene (**B14**). PAINS groups were detected only in the structure of **B7**, **B9**, and **CIP** (Table 3).

It can be observed that only five Schiff bases (**B6**–**9** and **B14**) and **CIP** have good predictions from GSK 4/400 rules, which say that substances with clogP < 4 and a molecular weight <400 Da have better drug-like properties [46]. Schiff bases, due to their hydrazone moiety, may have, according to the MedChem rules [47], potentially reactive or promiscuous behavior and also received a warning from the Pfizer 3/75 rule (substances with clogP > 3 and tPSA < 75 Å^2^ are more likely to have in vivo toxicity), due to the likelihood of promiscuous binding [48].

#### 2.2.2. Molecular Docking

The DNA gyrase is built of two A subunits (gyrA) and two B subunits (gyrB) that form an A2B2 heterotetramer. Topoisomerase IV consists of two constitutive subunits: parE (homologous to DNA gyrase subunit B–gyrB) and parC (homologous to DNA gyrase subunit A–gyrA) [6].

Fluoroquinolones act by inhibiting the gyrA subunit and also as competitive inhibitors of the ATP-binding site on the gyrB subunit [5].

The best antibacterial activity for our Schiff bases was displayed against the Gram-positive *Listeria monocytogenes*. In this view, we selected as docking targets the two DNA gyrases (DNA gyrase subunit A–gyrA and DNA gyrase subunit B–gyrB) from *L. monocytogenes*, since these are validated drug targets, currently used in drug design [5,21,42].

**Figure 2:**
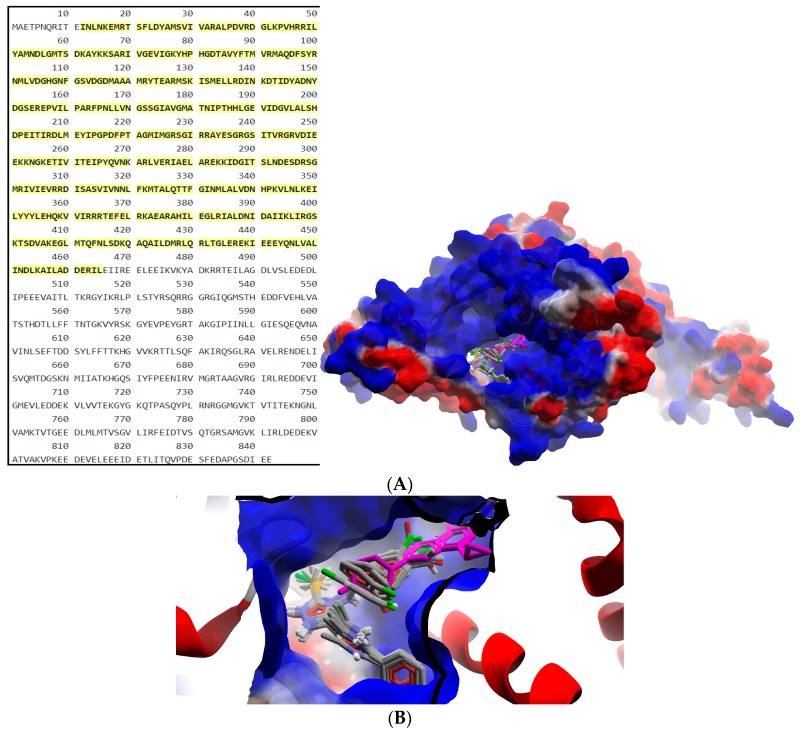
General view (**A**) and detail (**B**) of the best docking poses of ligands against gyrA. Target is depicted as thin sticks with a secondary structure drawn as a cartoon backbone and semi-transparent electrostatic molecular surface (cropped in the detailed view), where ligands are figured as ball-and-stick (Schiff bases are CPK colored, meanwhile **CIP** is pink-magenta).

**Figure 3:**
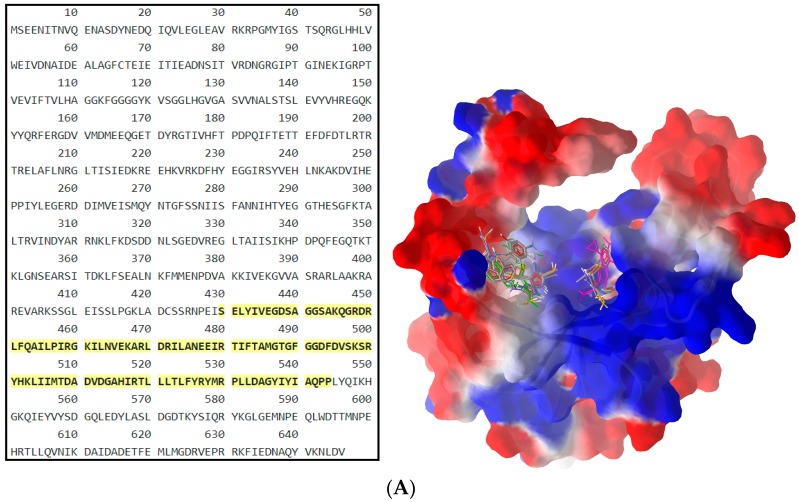

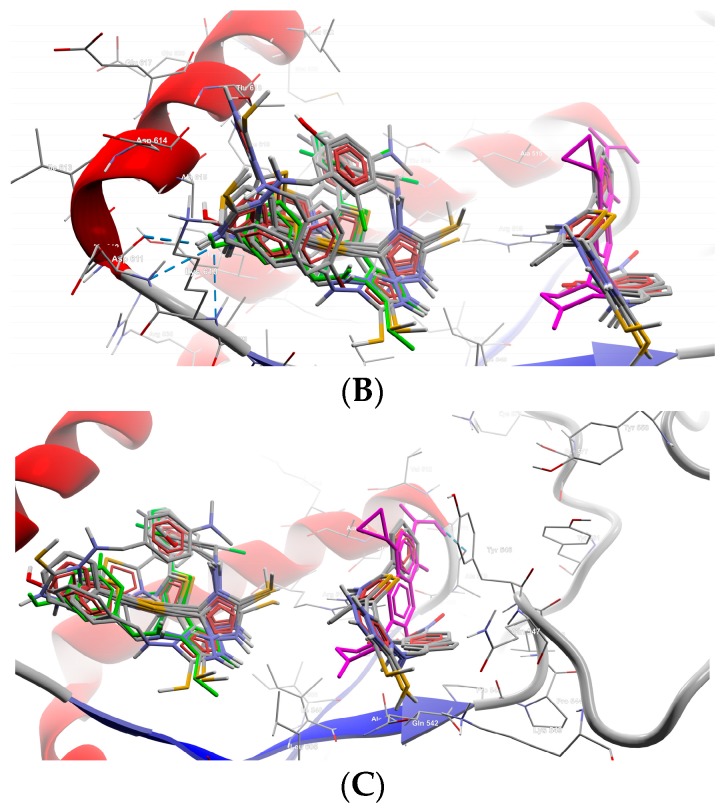
General view (**A**) and details (**B**,**C**) of the best docking poses of ligands against gyrB. Target is depicted as thin sticks with a secondary structure drawn as a cartoon backbone and semi-transparent electrostatic molecular surface (cropped in the detailed view), where ligands are figured as ball-and-stick (**CIP** is pink-magenta, **B8** is green, meanwhile the rest of the Schiff bases are CPK colored). In the general view (**A**), the right group is made from **CIP**, **B10**, **B11**, and **B13**; meanwhile, the left group is made of the rest of the Schiff bases (including here **B8**); Detail (**B**) shows the left group (**B8** group), image being focused on **B8** (green) binding mode, emphasizing the three H-bonds established with Lys610 and Asp611 (2 H-bonds) (cropped view showing only the nearest AAs residues–until a 7.5 Å distance from **B8**); Detail (**C**) shows the right group (**CIP** group), image being focused on the **CIP** (pink-magenta) binding mode, emphasizing the H-bond established with Ala510 (cropped view showing only the nearest AAs residues–until a 7.5 Å distance from **CIP**).

The results of the two molecular docking runs are presented briefly in Table 4 and Table 5, in terms of binding affinity (BA) for the best docking poses–using as scoring criteria a root-mean-square deviation equal to zero. A graphical depiction of the docking results is illustrated in Figure 2 and Figure 3. Detailed binding patterns and the total energetic interactions are shown in Table S2.

From Table 4, it can be easily observed that all Schiff bases are stronger binders than **CIP** (used as the control inhibitor) to gyrA; meanwhile, on gyrB, all compounds are considerably weaker binders. For **CIP**, the docking results are consistent with the data included in DrugBank [49,50]. From the supplementary data (Table S2), it can be observed that Schiff bases have a common binding pattern (also illustrated in Figure 2) against gyrA within the TOP4c domain (located between positions 12–465 in *Listeria monocytogenes*, according with the UniProtKB ID: Q8YAV6), in the region described between Phe88 and Lys270; meanwhile, **CIP** binds slightly differently, between Gly41 and Gln267. The TOP4c domain or DNA topoisomerase, type IIA, subunit A/C-terminal domain, has been described as having DNA-binding activity (according to Simple Modular Architecture Research Tool–SMART accession number: SM00434) [51,52]. In medallions, the canonical sequence of gyrA with the TOP4c domain (positions 12–465) (Figure 2A) and the canonical sequence of gyrB with the Toprim domain (positions 430–544) (Figure 3A), marked with yellow, are depicted.

**Figure 4:**
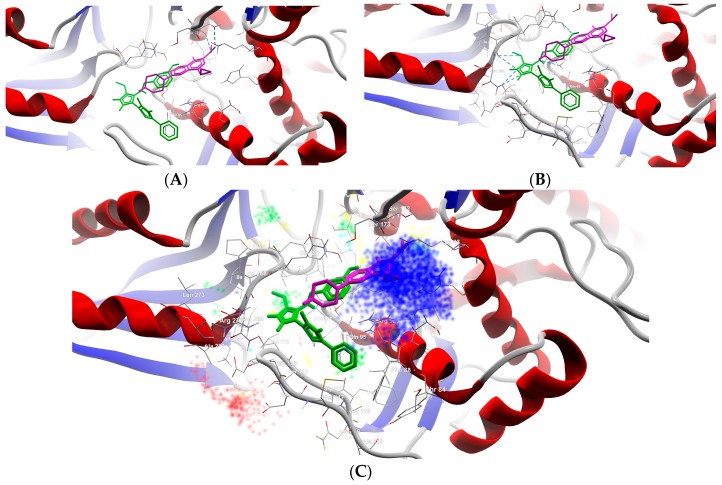
Details of the best docking poses of **CIP** (pink-magenta) and **B8** (green) against gyrA (target is depicted as thin sticks with a secondary structure drawn as a cartoon backbone, where ligands are figured as sticks and H-bonds are depicted as dashed blue lines). In detail (**A**), the image is focused on the **CIP** binding mode, emphasizing the two H-bonds established with Ser172 and Gly171 (in order to simplify the image, only the nearest AAs residues–until a 5.0 Å distance from **CIP**, are shown); In detail (**B**), the image is focused on the **B8** binding mode, emphasizing the six H-bonds established with Val113 (2 H-bonds), Val268 (2 H-bonds), Ser98, and Tyr266 (to simplify the image are shown only the nearest AAs residues–until a 5.0 Å distance from **B8**); Detail (**C**) illustrates the energy grid: green–steric favorable; light blue–hydrogen acceptor favorable; yellow–hydrogen donor favorable; red and dark blue–electrostatic interactions (cropped view showing only the nearest AAs residues–until a 7.5 Å distance from **B8**).

**Figure 5:**
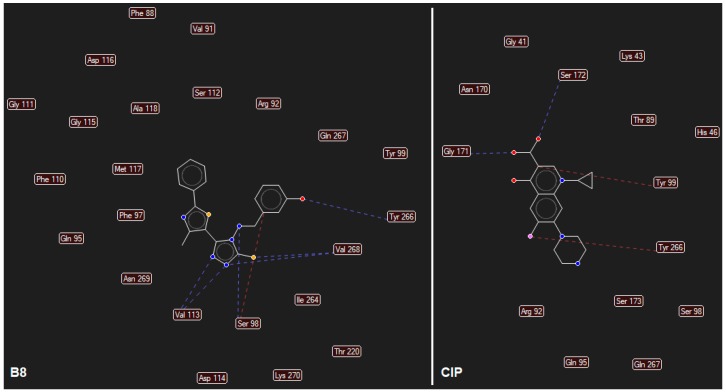
Mapping of H-bonds and steric interactions between ligands (case of **B8** and **CIP**) and gyrA (H-bonds are illustrated as blue dashed lines, strong steric interactions are represented as red dashed lines, the weak steric interaction are not figured, only the corresponding AAs are depicted).

**CIP** formed two H-bonds, between the carboxyl group from position 3 (O24, O25) with Gly171 and Ser172 of the TOP4c domain (Figure 4A). All Schiff bases formed at least three H-bonds between the azomethine nitrogen (N21) and serine (Ser98) and between the triazole nitrogens (N2 and N4) and the valine residues (Val113, Val268). Additional H-bonds are formed by **B7** (with Gln95), **B8** (with Tyr266–Figure 4B, Figure 5), **B10** (with Tyr266 and Gln267), and **B13** (with Tyr266). Even though the Schiff bases and ciprofloxacin bind differently to the target, but in the same subunit A (C-terminal domain of DNA topoisomerase IIA, (TOP4c)), both binding patterns competitively block the access of *O*-(5′-phospho-DNA)-tyrosine intermediate at its binding site, located in position 123 (according UniProtKB ID: Q7BSI9), in *Listeria monocytogenes*. This confirms the importance of the imine functional group for the binding mode of the Schiff bases.

Analyzing the docking results (Table 5), it can be observed that all screened compounds are weaker binders of gyrB than of gyrA. Moreover, the binding patterns indicate the lack of pharmacological relevance since the interacting region is placed partially outside the Toprim domain (Table S2, Figure 3B,C). Moreover, the H-bonds are scarce, and only **B1**, **B5**, **B7**–**11**, **B13**, **B15**, and **CIP** are able to establish such a type of interactions with gyrB (Table S2).

Analyzing the docking output, respectively, focusing on the best binders to gyrA, combined with the ADMET profiling, the best drug-like candidates are **B6**–**9** and **B14**, which comply with the GSK 4/400 rule (**CIP** also falls in the same category). Moreover, two of these drug candidates (**B8** and **B14**) are also free of PAINS, which make them the most balanced compounds, in terms of potency (being more potent than **CIP**) vs. safety (lacking of PAINS, meanwhile **CIP** being classified as an intermediate compound in terms of safety concerns).

**B8** is a stronger binder than **B14** (Table 4), interacting with the TOP4c domain of gyrA in a binding pocket described between Phe88 and Lys270 (Table S2, Figure 4 and Figure 5).

From Figure 4 and Figure 5, and data presented in Table S2, it can be observed that compound **B8** forms six H-bonds with gyrA (one between azomethine N21 and Ser98, two between Val113 and the triazolic nitrogens N2 and N4, two between Val268 and triazolic N4 and thiolic S32, and one between hydroxy-phenol substituent from meta position and Tyr266), and multiple steric interactions with surrounding AAs.

The competitive binding pattern of **B8** prevents both ATP and DNA binding to gyrA, with the formation of *O*-(5′-phospho-DNA)-tyrosine intermediate in the active site (located at position 123) being competitively blocked, resulting in the inhibition of its GO functions: ATP binding function (GO:0005524), ATP-hydrolyzing activity (GO:0003918), and DNA binding (GO:0003677). Such a binding pattern prevents the topological transformation of bacterial DNA. Due to the homology of the subunit A (gyrA) of the bacterial DNA gyrase and parC domain of topoisomerase IV, it is also expected that the GO functions of topoisomerase IV will be inhibited.

## 3. Materials and Methods

### 3.1. Antibacterial Activity Assay

#### 3.1.1. Determination of the Inhibition Zone Diameters

The in vitro antimicrobial activity was evaluated using the cup-plate agar diffusion method, according to the Clinical and Laboratory Standards Institute (CLSI) guidelines [53]. For the antibacterial testing, Mueller-Hinton agar medium was used. The cell density was adjusted to the density of a 0.5 McFarland standard. A volume of 20 µL of each compound solution (5 mg/mL in dimethyl sulfoxide-DMSO) was delivered into the wells (100 µg/well). Ciprofloxacin (100 µg/well) was used as the standard drug. The controls were performed with sterile broth, overnight culture, and 20 µL of DMSO. The plates were incubated at 35 °C. Zone diameters were measured after 24 h. Tests were repeated three times. The solvent used for the stock solutions (5 mg/mL), DMSO (Merck, Germany), did not express inhibitory activity against the tested bacterial strains.

#### 3.1.2. Calculation of the Percentage Activity Index (% AI)

Percentage activity index (% AI) was determined using the mathematical formula [54]:

% AI = (Zone of the inhibition of synthetic compound/Zone of the inhibition of reference drug) × 100

#### 3.1.3. Determination of MIC and MBC Values

The microorganisms used for the antimicrobial activity evaluation were obtained from the University of Agricultural Sciences and Veterinary Medicine Cluj-Napoca, Romania. The Gram-positive bacteria (*Staphylococcus aureus* ATCC 49444, *Listeria monocytogenes* ATCC 19115) and Gram-negative bacteria (*Pseudomonas aeruginosa* ATCC 27853, *Salmonella typhimurium* ATCC 14028) were maintained on plate count agar slants, at 4 °C. The cultures were maintained on Mueller Hinton agar (bioMérieux, Marcy l’Etoile, France). The bacteria were cultured overnight in 5 mm Mueller Hinton broth (bioMérieux, Marcy l’Etoile, France) in a shaker incubator (Heidolph Inkubator 1000 coupled with Heidolph Unimax 1010, Germany), at 37 °C, 150 rpm, until the culture reached an OD_550_ of 0.02 (Nanodrop Spectophotometer ND-1000, USA), corresponding to 10^8^ CFU mL^−1^. Before the incubation with materials, the cultures were diluted to 10^5^ CFU mL^−1^.

Stock solutions (1 mg/mL) were prepared by dissolving the test compounds and the reference antibiotic (ciprofloxacin), respectively, in sterile DMSO. These solutions were stored at 4 °C. Series of double diluting solutions of the above compounds were prepared in RPMI 1640 medium, obtaining final concentrations in the range of 500 µg/mL to 0.015 µg/mL.

The broth microdilution method was employed for the minimum inhibitory concentration (MIC) test [53]. The growth control, sterility control, and control of antibacterial compounds were used. Plates were incubated at 37 °C, for 24 h, and next, MICs were determined, by adding resazurin (20 µL, 0.02%), followed by a 2 h incubation. For determining the minimum bactericidal concentration (MBC), a 0.01 mL aliquot of the medium drawn from the culture tubes, showing no macroscopic growth after 24 h, was subcultured on nutrient agar/potato dextrose agar plates, to determine the number of the vital organisms and was incubated further at 37 °C, for 24 h. All MIC and MBC tests were repeated three times.

### 3.2. Virtual Screening

#### 3.2.1. ADMET Predictions

FAF-Drugs4 [55,56] was used to screen all ligands in order to predict their ADME-Tox properties. The input files (previously generated SDF files) were formatted according to FAF-Drugs4’s requirements using Bank-Formatter [55]. XLOGP3 [54] was chosen as the logP computation program to estimate lipophilicity and the derived ADMET descriptors. ADMET screening was carried out using a series of FAF-Drugs4’s built-in filters for drug-likeness. The *Drug-Like Soft filter* of FAF-Drugs4 is based on the physicochemical and molecular properties and the bioavailability rules used widely for drug discovery [57,58,59,60,61]. The *Drug-Like Soft filter* uses a built-in statistical analysis of drugs [55] extracted from the *e-Drugs3D library* [62] for the threshold values of computed descriptors. Additional filters were used for the detection of the non-peptidic inhibitors of protein-protein interactions (PPIs) [63], the detection of the undesirable moieties and substructures (UMSs) involved in toxicity problems [46,63,64,65], covalent inhibitors (CIs) [32,33], and Pan-Assay Interference Compounds (PAINS) [66,67]. The detection of PAINS was done using a set of three filters [55,67,68]. Finally, a series of ADMET filters, currently used by pharmaceutical companies, were used to assess the safety profiling: MedChem rules [47], the GSK 4/400 rule [46], the Pfizer 3/75 rule [48], and the estimation of phospholipidosis induction (PPDI) [69]. The MedChem is a package of 275 rules developed by Eli Lilly and Company (Indianapolis, IN, USA) to identify compounds that may interfere with the biological assays–we chose in our VS run a 100-demerit cutoff (the regular setting of FAF-Drugs4).

#### 3.2.2. Molecular Docking

The molecular docking was performed on DNA gyrase subunit A–gyrA and DNA gyrase subunit B–gyrB from *L. monocytogenes*. A cross-search between The Universal Protein Resource–UniProt [70] and RCSB Protein Data Bank–RCSB-PDB [71] did not reveal any experimental structure for DNA gyrases from *L. monocytogenes*; consequently, there were constructed homologue models for both of them, using SWISS-MODEL [72], via the ExPASy web server [73].

The previously optimised Tripos MOL2 files of corresponding Schiff bases were docked against each of the two gyrases, in two separate runs, with PyRx–Python Prescription 0.9.5 [74] using AutoDock Vina [75] as the docking algorithm. AutoDock Vina is able to automatically calculate the grid maps and use an X-score inspired scoring function [76] to predict the noncovalent binding of ligands and to cluster the results. Since Autodock Vina is able to automatically calculate the grid maps, both runs were performed as blind docking [77] in order to detect all the possible binding sites and binding patterns. The exhaustiveness of each docking run was set to 80 (increased 10 times from the default value of software), in order to improve the accuracy of the predictions [75,78]. Supplementary, Molegro Molecular Viewer v2.5–MMV v2.5 (Molegro, A CLC Bio Company, Aarhus N, Denmark) was used for data extraction (binding patterns, energy contribution) and high-resolution renderings.

## 4. Conclusions

Fifteen thiazolyl-triazole Schiff bases, previously synthesized, have been investigated for their antibacterial potential, against Gram-positive and Gram-negative bacterial strains. The determination of the inhibitory zone diameters showed that compounds **B1**, **B2**, **B9**, and **B10** were the most potent against Gram-positive *L. monocytogenes*, with an equal or a superior effect (**B10**), compared to ciprofloxacin. MICs and MBCs were in agreement with the results obtained. Regarding the activity against the Gram-negative strains, most of the compounds inhibited the growth of *P. aeruginosa*, but MICs were smaller than the reference (**B5**, **B6**, **B11**–**15**) or equal to ciprofloxacin (**B9**). The calculated MBC/MIC ratio suggested a bactericidal effect for the new molecules.

The molecular docking study, performed on DNA-gyrA and gyrB from *L. monocytogenes*, revealed that the thiazolyl-triazole Schiff bases have a common binding pattern to gyrA and competitively block the access of *O*-(5′-phospho-DNA)-tyrosine intermediate at its binding site. All Schiff bases make at least three H-bonds between the azomethine nitrogen, the triazole nitrogens (N2 and N4), respectively, with AA residues from the TOP4c domain of gyrA. All screened compounds are weaker binders to gyrB than gyrA and the binding patterns indicate the lack of pharmacological relevance, since the interacting region is placed partially outside the Toprim domain.

The ADMET profiling revealed that all Schiff bases are non-inducers of phospholipidosis. The virtual screening selected the thiazolyl-triazole derivative **B8** as the best *drug-like* candidate, which complied with the safety rules (GSK 4/400, PAINS), and was more efficient than **CIP**, on gyrA. Plus, its binding pattern prevents both ATP and DNA binding to gyrA, and consequently, prevents the topological transformation of bacterial DNA.

All data collected from the in vitro antibacterial evaluation and the virtual screening, may be considered as a structural basis for the design of new antibacterial drugs, acting as DNA-gyrase inhibitors, without severe side effects.

## Figures and Tables

**Table 1:** The antibacterial activity of compounds **B1**–**15**.

Cp.	Gram-Positive Bacteria				Gram-Negative Bacteria					
	*Staphylococcus aureus* ATCC 25923		*Listeria monocytogenes* ATCC 35152		*Escherichia coli* ATCC 25922		*Salmonella typhimurium* ATCC 13311		*P. aeruginosa* ATCC 27853	
	Diameter (mm)	%AI	Diameter (mm)	%AI	Diameter (mm)	%AI	Diameter (mm)	%AI	Diameter (mm)	%AI
**B1**	14	50	**18**	**100**	14	51.8	16	72.7	19	73
**B2**	14	50	**18**	**100**	14	51.8	18	81.8	19	73
**B3**	14	50	16	88.8	14	51.8	18	81.8	16	61.5
**B4**	14	50	14	77.7	14	51.8	18	81.8	18	69.2
**B5**	14	50	14	77.7	14	51.8	18	81.8	21	80.7
**B6**	14	50	14	77.7	14	51.8	16	72.7	21	80.7
**B7**	16	57.1	12	66.6	14	51.8	16	72.7	18	69.2
**B8**	12	42.8	12	66.6	14	51.8	16	72.7	18	69.2
**B9**	14	50	**18**	**100**	16	59.2	16	72.7	20	76.9
**B10**	**18**	**64.2**	**20**	**111.1**	16	59.2	18	81.8	18	69.2
**B11**	12	42.8	8	44.4	16	59.2	18	81.8	21	80.7
**B12**	12	42.8	14	77.7	14	51.8	18	81.8	21	80.7
**B13**	12	42.8	12	66.6	14	51.8	18	81.8	21	80.7
**B14**	12	42.8	16	88.8	16	59.2	18	81.8	21	80.7
**B15**	16	57.1	10	55.5	14	51.8	18	81.8	21	80.7
**CIP**	**28**	**100**	**18**	**100**	**27**	**100**	**22**	**100**	**26**	**100**

The values obtained for the most active compounds are marked in bold. Cp.: Compounds; **CIP**: ciprofloxacin; %AI = percentage activity index ((Zone of the inhibition of synthetic compound/Zone of the inhibition of reference drug) × 100).

**Table 2:** Minimum Inhibitory Concentration (MIC) and Minimum Bactericidal Concentration (MBC) (in µg/mL) of compounds **B1**–**15**.

Cp.	*S. aureus* ATCC 49444		*L. monocytogenes* ATCC 19115		*P. aeruginosa* ATCC 27853		*S. typhimurium* ATCC 14028	
	MIC	MBC	MIC	MBC	MIC	MBC	MIC	MBC
**B1**	31.25	31.25	**1.95**	**3.9**	7.81	15.62	62.5	125
**B2**	31.25	31.25	3.9	7.8	7.81	15.62	62.5	62.5
**B3**	62.5	62.5	3.9	7.8	15.62	31.25	62.5	62.5
**B4**	31.25	62.5	3.9	7.8	7.81	15.62	62.5	62.5
**B5**	31.25	31.25	**1.95**	**3.9**	**1.95**	**3.9**	62.5	62.5
**B6**	31.25	62.5	**1.95**	**3.9**	**1.95**	**3.9**	62.5	62.5
**B7**	31.25	31.25	**1.95**	**3.9**	7.81	15.62	62.5	125
**B8**	62.5	62.5	3.9	**3.9**	7.81	15.62	62.5	125
**B9**	31.25	31.25	**1.95**	**3.9**	3.9	7.8	62.5	125
**B10**	31.25	31.25	3.9	7.8	7.81	15.62	62.5	62.5
**B11**	62.5	62.5	**1.95**	**3.9**	**1.95**	**3.9**	62.5	62.5
**B12**	31.25	62.5	**1.95**	**3.9**	**1.95**	**1.95**	62.5	125
**B13**	31.25	62.5	**1.95**	**3.9**	**1.95**	**3.9**	31.25	62.5
**B14**	**15.62**	31.25	**1.95**	**3.9**	**1.95**	**3.9**	62.5	125
**B15**	31.25	62.5	**1.95**	**3.9**	**1.95**	**3.9**	62.5	62.5
**Ciprofloxacin**	**1.95**	**3.9**	**3.9**	**7.8**	**3.9**	**7.8**	**0.97**	**1.95**
**Inoculum Control**	+++		+++		+++		+++	
**Broth control**	No growth		No growth		No growth		No growth	

Cp.: Compounds; +++ Indicates growth in all concentrations. The values obtained for the most active compounds are marked in bold.

**Table 3:** ADMET profiling–risks and safety concerns.

Cp.	MW (Da)	logP	tPSA (Å^2^)	PPIs	UMSs	CIs	PAINS Filters			PPDI	Med Chem	GSK 4/400	Pfizer 3/75
							A	B	C				
**B1**	446.38	5.69	104.1	Yes	thiol hal.	thiol	ND	ND	ND	NI	hydr.	bad	warn.
**B2**	446.38	5.69	104.1	Yes	thiol hal.	thiol	ND	ND	ND	NI	hydr.	bad	warn.
**B3**	446.38	5.69	104.10	Yes	thiol hal.	thiol	ND	ND	ND	NI	hydr.	bad	warn.
**B4**	411.93	5.06	123.00	Yes	thiol hal.	thiol	ND	ND	ND	NI	hydr.	bad	warn.
**B5**	456.38	5.13	123.00	Yes	hal.	thiol	ND	ND	ND	NI	hydr.	bad	warn.
**B6**	395.48	4.53	123.00	Yes	thiol hal. F	thiol	ND	ND	ND	NI	hydr.	good	warn.
**B7**	393.49	4.08	143.23	Yes	thiol phenol	thiol	I^479^ I^479b^	ND	ND	NI	hydr.	good	warn.
**B8**	393.49	4.08	143.23	Yes	thiol phenol	thiol	ND	ND	ND	NI	hydr.	good	warn.
**B9**	393.49	4.08	143.23	Yes	thiol phenol	thiol	I^215^	ND	ND	NI	hydr.	good	warn.
**B10**	422.48	4.26	149.92	Yes	thiol nitro	thiol	ND	ND	ND	NI	hydr.	bad	warn.
**B11**	422.48	4.26	149.92	Yes	thiol nitro	thiol	ND	ND	ND	NI	hydr.	bad	warn.
**B12**	407.51	4.41	132.23	Yes	thiol	thiol	ND	ND	ND	NI	hydr.	bad	warn.
**B13**	407.51	4.41	132.23	Yes	thiol	thiol	ND	ND	ND	NI	hydr.	bad	warn.
**B14**	383.51	4.45	151.24	Yes	thiol thp.	thiol	ND	ND	ND	NI	hydr.	good	warn.
**B15**	420.55	4.56	126.24	Yes	thiol	thiol	ND	ND	ND	NI	hydr.	bad	warn.
**CIP**	331.34	0.28	81.98	Not	hal. F	ND	I^215^	ND	ND	NI	ND	good	bad

Cp.: Compounds; MW: molecular weight; logP: logarithm of compound partition coefficient between n-octanol and water; tPSA: topological polar surface area; PPIs: protein-protein interactions; UMSs: undesirable moieties and substructures; hal.: halogenure; hal. F: halogenure with Fluorine; thp.: thiophene; CIs: covalent inhibitors; PAINS: Pan-Assay Interference Compounds; ND: none detected (compound is free of problematic sub-structures for the corresponding risk criteria). I^479^: intermediate compound which embeds a low-risk structural PAINS alert with a number of occurrences below the threshold, according to the PAINS filter more150_hzone_phenol_A. I^479b^: intermediate compound which embeds a low-risk structural PAINS alert with a number of occurrences below the threshold, according to the PAINS filter more150_hzone_phenol_A_bis. I^215^: intermediate compound which embeds a low-risk structural PAINS alert with a number of occurrences below the threshold, according to the PAINS filter more150_hzone_phenol_B. PPDI: phospholipidosis induction; NI: non-inducer of phospholipidosis; hydr.: hydrazine; warn.: warning (have to be used with caution as blindly applying such recipes can discard from development many interesting molecules)

**Table 4:** Predicted binding affinity, interaction domain, and polar interactions between compounds **B1**–**15** and the DNA gyrase A from *Listeria monocytogenes*.

Backbone of the Compounds B1–15	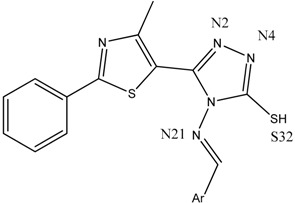		
Compound	gyrA BA (kcal/mol)	Atom ID of Ligand	Interacting AA Residue
**B1**	−8.9	N (2)	Ser112
		N (4)	Val113
		N (21)	Ser98
**B2**	−7.6	N (2)	Ser112, Val113
		N (4)	Val113
		N (21)	Ser98
		S (32)	Val268
**B3**	−8.1	N (2)	Val113
		N (4)	Val113, Val268
		N (21)	Ser98
		S (32)	Val268
**B4**	−8.5	N (2)	Val113
		N (4)	Val113, Val268
		N (21)	Ser98
**B5**	−8.8	N (2)	Val113
		N (4)	Val113, Val268
		N (21)	Ser98
		S (32)	Val268
**B6**	−8.8	N (2)	Val113
		N (4)	Val113, Val268
		N (21)	Ser98
**B7**	−8.1	N (2)	Val113
		N (4)	Val113, Val268
		N (21)	Ser98
		Phenolic O	Gln95, Ser98
**B8**	−8.7	N (2)	Val113
		N (4)	Val113, Val268
		N (21)	Ser98
		Phenolic O	Tyr266
		S (32)	Val268
**B9**	−8.5	N (2)	Val113
		N (4)	Val113, Val268
		N (21)	Ser98
		S (32)	Val268
**B10**	−9.1	N (2)	Val113
		N (4)	Val113, Val 268
		N (21)	Ser98
		Nitro N	Tyr266
		Nitro O	Gln267
**B11**	−8.8	N (2)	Val113
		N (4)	Val113, Val268
		N (21)	Ser98
		S (32)	Val268
**B12**	−8	N (4)	Val113, Val268
		N (21)	Ser98
		Methoxy O	Ser98
		S (32)	Val268
**B13**	−8.8	N (2)	Val113
		N (4)	Val113, Val268
		N (21)	Ser98
		Methoxy O	Tyr266
**B14**	−7.9	N (2)	Val113
		N (4)	Val113, Val268
		N (21)	Ser98
		S (32)	Val268
**B15**	−8.8	N (2)	Val113
		N (4)	Val113, Val268
		N (21)	Ser98
**CIP**	−7.1	O (24)	Ser172
		O (25)	Gly171

**Table 5:** Predicted binding affinity, interaction domain, and polar interactions between compounds **B1**–**15** and the DNA gyrase B from *Listeria monocytogenes*.

Backbone of the compounds B1–15	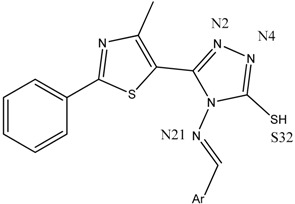		
Compound	gyrB BA (kcal/mol)	Atom ID of Ligand	Interacting AA Residue
**B1**	−6.3	N (4)	Asp611
		S (32)	Asp614
**B2**	−6.7	NA	NA
**B3**	−6.6	NA	NA
**B4**	−7.3	NA	NA
**B5**	−6.3	N (2)	Asp614, Thr618
**B6**	−7.2	NA	NA
**B7**	−6.3	N (4)	Asp611
		Phenolic O (30)	Thr618
		Thiazole S (16)	Asp614
**B8**	−7.3	Phenolic O (30)	Lys610, Asp611
**B9**	−7.2	N (21)	Asn608
		Phenolic O (30)	Asp611, Ala615
**B10**	−6.8	Nitro O (31,32)	Arg518
**B11**	−6.8	Nitro N (30)	Arg518
		Nitro O (32)	Arg518
		S (33)	Gln542
**B12**	−6.4	NA	NA
**B13**	−6.6	N (21)	Gln542
		Methoxy O (30)	Arg518
**B14**	−6.5	NA	NA
**B15**	−6.2	N (4)	Asp611
**CIP**	−6.5	O (25)	Ala510

## Supplementary Materials

Supplementary materials can be found at www.mdpi.com/1422-0067/19/1/222/s1.
