# Supplementary material for: Antibacterial Evaluation and Virtual Screening of New Thiazolyl-Triazole Schiff Bases as Potential DNA-Gyrase Inhibitors
Source: Int J Mol Sci. 2018 Jan 11;19(1):222. doi: 10.3390/ijms19010222 (PMC5796171; doi:10.3390/ijms19010222)
Supplement: Supplementary file 1 [file ijms-19-00222-s001.zip › ijms-250603-supplementary materials/ijms-250603-Table S1.docx]

**Table S1.** The MBC/MIC ratio for Schiff bases **B1**–**15**.

| **Compound** | **MBC/MIC** | | | |
| --- | --- | --- | --- | --- |
|  | ***S. aureus***  **ATCC 49444** | ***L. monocytogenes***  **ATCC 19115** | ***P. aeruginosa***  **ATCC 27853** | ***S. typhimurium***  **ATCC 14028** |
| **B1** | 1 | 2 | 2 | 2 |
| **B2** | 1 | 2 | 2 | 1 |
| **B3** | 1 | 2 | 2 | 1 |
| **B4** | 2 | 2 | 2 | 1 |
| **B5** | 1 | 2 | 2 | 1 |
| **B6** | 2 | 2 | 2 | 1 |
| **B7** | 1 | 2 | 2 | 2 |
| **B8** | 1 | 1 | 2 | 2 |
| **B9** | 1 | 2 | 2 | 2 |
| **B10** | 1 | 2 | 2 | 1 |
| **B11** | 1 | 2 | 2 | 1 |
| **B12** | 2 | 2 | 1 | 2 |
| **B13** | 2 | 2 | 2 | 2 |
| **B14** | 2 | 2 | 2 | 2 |
| **B15** | 2 | 2 | 2 | 1 |
